# FSP1 confers ferroptosis resistance in KEAP1 mutant non-small cell lung carcinoma in NRF2-dependent and -independent manner

**DOI:** 10.1038/s41419-023-06070-x

**Published:** 2023-08-26

**Authors:** Jong Woo Kim, Min-Ju Kim, Tae-Hee Han, Ji-Yoon Lee, Sangok Kim, Hyerin Kim, Kyoung-Jin Oh, Won Kon Kim, Baek-Soo Han, Kwang-Hee Bae, Hyun Seung Ban, Soo Han Bae, Sang Chul Lee, Haeseung Lee, Eun-Woo Lee

**Affiliations:** 1 Metabolic Regulation Research Center, Korea Research Institute of Bioscience and Biotechnology (KRIBB), Daejeon, 34141 Republic of Korea; 2 Department of Functional Genomics, University of Science and Technology (UST), Daejeon, 34113 Republic of Korea; 3 Department of Pharmacy, College of Pharmacy and Research Institute for Drug Development, Pusan National University, Busan, 46241 Republic of Korea; 4 Biotherapeutics Translational Research Center, Korea Research Institute of Bioscience and Biotechnology (KRIBB), Daejeon, 34141 Republic of Korea; 5 Department of Biomolecular Science, University of Science and Technology (UST), Daejeon, 34113 Republic of Korea; 6 Korea Bioinformation Center, Korea Research Institute of Bioscience and Biotechnology (KRIBB), Daejeon, 34141 Republic of Korea; 7 Biodefense Research Center, Korea Research Institute of Bioscience and Biotechnology (KRIBB), Daejeon, 34141 Republic of Korea; 8 Severance Biomedical Science Institute, Yonsei University College of Medicine, 50 Yonsei-ro, Seodaemun-gu, Seoul, 03722 Republic of Korea; 9 Severance Biomedical Science Institute, Graduate School of Medical Science, Brain Korea 21 Project, Yonsei University College of Medicine, Seoul, 03722 Republic of Korea; 10 School of Pharmacy, Sungkyunkwan University, Suwon, 16419 Republic of Korea

**Keywords:** Cell death, Non-small-cell lung cancer

## Abstract

Ferroptosis, a type of cell death induced by lipid peroxidation, has emerged as a novel anti-cancer strategy. Cancer cells frequently acquire resistance to ferroptosis. However, the underlying mechanisms are poorly understood. To address this issue, we conducted a thorough investigation of the genomic and transcriptomic data derived from hundreds of human cancer cell lines and primary tissue samples, with a particular focus on non-small cell lung carcinoma (NSCLC). It was observed that mutations in Kelch-like ECH-associated protein 1 (*KEAP1*) and subsequent nuclear factor erythroid 2-related factor 2 (NRF2, also known as NFE2L2) activation are strongly associated with ferroptosis resistance in NSCLC. Additionally, *AIFM2* gene, which encodes ferroptosis suppressor protein 1 (FSP1), was identified as the gene most significantly correlated with ferroptosis resistance, followed by multiple NRF2 targets. We found that inhibition of NRF2 alone was not sufficient to reduce FSP1 protein levels and promote ferroptosis, whereas FSP1 inhibition effectively sensitized *KEAP1*-mutant NSCLC cells to ferroptosis. Furthermore, we found that combined inhibition of FSP1 and NRF2 induced ferroptosis more intensely. Our findings imply that FSP1 is a crucial suppressor of ferroptosis whose expression is partially dependent on NRF2 and that synergistically targeting both FSP1 and NRF2 may be a promising strategy for overcoming ferroptosis resistance in cancer.

## Introduction

Ferroptosis is a form of regulated cell death (RCD) characterized by membrane rupture due to the accumulation of lipid peroxides in membrane phospholipids [1, 2]. Ferrous iron (Fe^2+^) is essential for lipid peroxidation and ferroptosis; Brent R. Stockwell and coworkers named this process “Ferroptosis” in 2012 [1, 3]. Glutathione peroxidase 4 (GPX4) primarily controls membrane lipid peroxide levels by converting lipid peroxide to lipid alcohol, using glutathione (GSH) as a cofactor [4, 5]. As a result, inhibition of GPX4 or GSH supply can lead to ferroptosis [1, 6]. The oxidation of polyunsaturated fatty acids (PUFAs) in membrane phospholipids is a hallmark of ferroptosis [2, 7]; therefore, enzymes that incorporate PUFAs into membrane phospholipids, such as acyl-CoA synthetase long-chain family member 4 (ACSL4) and lysophosphatidylcholine acyltransferase 3 (LPCAT3), are considered necessary for ferroptosis [8, 9]. Additionally, the PUFA biosynthesis and uptake pathways are critical factors in determining vulnerability of ferroptosis [10–12]. A reduced form of coenzyme Q_10_ (CoQ_10_H_2_) is a natural lipophilic radical-trapping agent (RTA) that prevents ferroptosis by directly eliminating lipid peroxyl radicals [13, 14]. Furthermore, increased expression of FSP1, previously known as apoptosis-inducing factor mitochondria-associated 2 (AIFM2), is associated with ferroptosis resistance because FSP1 is essential for recycling oxidized CoQ_10_ into reduced CoQ_10_H_2_ [13, 14]. Inhibition of FSP1 by iFSP1, an inhibitor of FSP1, has been shown to promote ferroptosis in various types of cancer cells, indicating a general role for FSP1 in ferroptosis suppression [14]. A recent study has revealed that FSP1 is also responsible for recycling vitamin K and contributes to ferroptosis resistance [15].

Lung cancer is classified as either small-cell lung carcinoma (SCLC) or non-small cell lung carcinoma (NSCLC), with NSCLC accounting for 80% of all lung cancer cases [16, 17]. Common mutations in NSCLC include those in Kirsten rat sarcoma virus (*KRAS*), epidermal growth factor receptor (*EGFR*), tumor protein p53 (*TP53*), and Kelch-like ECH-associated protein 1 (*KEAP1*), and attempts have been made to target these mutations in cancer therapy [18–21]. Among these, *KEAP1* mutations result in the constitutive activation of nuclear factor erythroid 2-related factor 2 (NRF2, also known as NFE2L2), which has been associated with tumor malignancy and drug resistance, leading to poor prognosis [22]. NRF2 is therefore an attractive target for *KEAP1*-mutant tumors [23–25]. Mechanistically, KEAP1 binds to NRF2 and promotes its degradation via the KEAP1-CUL3 E3 ubiquitin ligase complex under normal conditions [26]. When reactive oxygen species (ROS) levels increase in the cell, they react with the cysteine sensors of KEAP1, triggering a conformational change in KEAP1, which in turn releases NRF2 [27–29]. Released NRF2 translocates into the nucleus and activates NRF2 target genes containing antioxidant response elements (ARE) to reduce ROS [29]. Given that ARE genes regulate multiple metabolic pathways, including iron metabolism, GSH metabolism, thioredoxin metabolism, and NADPH production, all of which are crucial in ferroptosis, NRF2 is regarded as a master regulator of ferroptosis in various types of cancer cells [26, 30–32]. However, despite numerous studies supporting NRF2’s critical roles in the regulation of ferroptosis, it remains unclear which NRF2 target genes specifically or preferentially inhibit ferroptosis.

In this study, we attempted to elucidate key NRF2 target genes mostly associated with ferroptosis susceptibility by investigating the genomic and transcriptomic data derived from both cancer cell lines and primary tissues. Our findings revealed that elevated expression levels of several NRF2 target genes are linked to increased resistance to ferroptosis, with FSP1 emerging as the most significant contributor. Nevertheless, knockdown of NRF2 was insufficient to completely reduce the protein levels of FSP1 and did not significantly promote ferroptosis. However, direct inhibition of FSP1 was effective in enhancing ferroptosis, and NRF2 inhibition in the presence of an FSP1 inhibitor further facilitated ferroptosis. Our study suggests that the expression of FSP1 is NRF2-dependent, but the mechanisms regulating their ferroptosis sensitivity are independent. As a result, dual-targeted therapy with FSP1 and NRF2 may be an attractive strategy for the treatment of KEAP1-mutant NSCLC.

## Materials and methods

### Collection of cancer cell line and primary tissue datasets

Datasets of human cancer cell lines were acquired from the DepMap portal (https://depmap.org/portal/, version 22Q2), which includes comprehensive genomic, transcriptomic, proteomic, and drug response information for over 1000 cell lines derived from more than 30 cancer types. Sample_info, CCLE_mutations, CCLE_RNAseq_reads/CCLE_expression, and proteomics files were used to access the data on cancer type/lineage, gene mutations, and gene and protein expression levels, respectively. In addition, data on drug responses to ferroptosis inducers (FINs), including RSL3, ML162, ML210, and erastin, were obtained from the drug sensitivity AUC (CTD^2) file. The area under the curve (AUC) of dose-response viability was used as a measure of drug sensitivity, with a lower AUC indicating greater drug sensitivity. This study utilized 1,317 cancer cell lines with transcriptome, proteome, and drug sensitivity data, excluding cells derived from blood cancer. The mRNA and protein expression levels of the gene were reported using log2-transformed transcript per million (TPM) values with a pseudo-count of one and normalized protein quantitation levels [33], respectively.

RNA-seq data of human cancer patients was obtained from the TCGA legacy gene expression data via the GDCquery function in the R package TCGAbiolinks. The data contained read counts and TPM values for 19,121 protein-coding genes in 9187 patient samples across 33 cancer types. Information on genetic alterations in individual patients or samples was obtained from the cBioPortal (https://www.cbioportal.org/). We excluded samples derived from the blood lineage.

### Identification of gene mutations associated with FIN sensitivity

To identify genetic alterations that were significantly enriched in FIN-resistant cells, we conducted an association analysis between drug response and mutational status for 16,616 genes with mutations observed in cancer cell lines. For each FIN, the cell lines were divided into two groups, sensitive (S) and resistant (R), based on the median AUC for the FIN. A contingency table was constructed for each gene to represent the number of mutant and wild-type cells in each group (S, R). A hypergeometric test was performed using a contingency table to assess the degree of significant enrichment of R cell lines in mutant cell lines. The *P*-value obtained from the test was subsequently transformed into −log_10_(*p*-value) to score each gene.

### Functional enrichment analysis

Gene Set Enrichment Analysis (GSEA) was performed to identify pathways significantly enriched in differentially expressed genes between groups (e.g., *KEAP1* mutation vs. wild-type). Differential gene expression analysis was conducted by the Wald test implemented in the R package DESeq2, yielding a ranked list of genes based on Wald statistics. The WikiPathways database was utilized for functional annotation and obtained via the R package msigdbr. Of the 664 available gene sets, 627 were retained for further investigation, excluding 37 sets named with disease terms. GSEA was performed via the R package fgsea with the following parameters: minSize, 10; maxSize, 500; and nperm, 100,000. The enrichment score for each pathway was defined as the product of −log10(q-value) and the sign of NES, where *q-*value indicates the false discovery rate-adjusted *p*-value and NES stands for the normalized enrichment score.

### Reagents

RSL3 (S8155) and Fer-1 (S7243) were purchased from Selleck Chemicals (USA). iFSP1 (HY-136057) was purchased from MedChemExpress (USA). C-11 BODIPY 581/591 (Invitrogen^TM^, D3861) and L-Glutamine solution 200 mM (Gibco^TM^, 25030081) were purchased from Thermo Fisher Scientific (Waltham, MA, USA). L-Methionine (M5308) and L-Cystine (C7602) were purchased from Sigma-Aldrich (USA). All dissolved reagents were stored at −20 °C until use.

### Cell culture

All NSCLC cells were obtained from Dr. Hyun-Seung Ban’s Lab. NCI-H1299, A549, and NCI-H460 cells were maintained in RPMI-1640 (Corning, 10-040-CVRC) supplemented with 10% fetal bovine serum (FBS) (Thermo, 10099141) and 1% antibiotic-antimycotic (Anti-Anti) (Gibco, 15240062). NCI-H2009, NCI-H1437, and NCI-H322 cells were maintained in Dulbecco’s modified Eagle’s medium (DMEM) (Hyclone, SH30243.01) containing 5% FBS and 1% Anti-Anti. NCI-H23 cells were maintained Hyclone DMEM containing 10% FBS and 1% Anti-Anti. All cells were incubated at 37 °C, 5% CO_2_, and humidified environment.

### Cysteine deprivation

For cysteine deprivation, DMEM without glutamine, methionine, or cysteine (Gibco, 21013024) was used. To exclude the amino acids contained in FBS, dialyzed FBS (dFBS) (Gibco, 26400-044) was used. Methionine powder was diluted in deionized distilled water and cysteine powder was diluted in 1 N HCl (Junsei, 20010S0350). Cysteine-deficient medium was composed of 10% dFBS, 1% Anti-Anti, 4 mM glutamine, and 0.2 mM methionine. As a positive control, 0.2 mM cysteine was added to cysteine-deficient medium. Following removal of the cell culture medium, the cells were washed twice with prewarmed Dulbecco’s phosphate-buffered saline (DPBS). The medium was then replaced with cysteine-deficient medium and cysteine-containing medium, treating simultaneously with iFSP1 or DMSO; the cells in the replaced medium were incubated for 24 h.

### Cell viability and lactate dehydrogenase assays

The cells were seeded at 2 × 10^4^/well in 48-well cell culture plates. Cell viability was measured using the CellTiter-Glo® 2.0 Cell Viability Assay reagent (Promega, G9243), and the experiment was carried out in accordance with the manufacturer’s protocol. The lactate dehydrogenase (LDH) release assay was performed using a Cytotoxicity Detection Kit (Roche, 11644793001). Calculations of LDH release were performed according to the manufacturer’s protocol.

### Lipid peroxidation assay

C-11 BODIPY 581/591 was used to measure lipid peroxidation. The cells were seeded at 1 × 10^5^ /well in 12-well cell culture plates. The following day, 45 min after RSL3 and iFSP1 treatment, 2.5 μM of C-11 BODIPY was added, and then the cells were incubated for 15 min at 37 °C. Cells were detached using trypsin-EDTA in a 1.5 mL microfuge tube and centrifuged at 326×*g* for 3 min. The cell pellet was then washed twice with cold DPBS. After resuspending the cells in 300 μL of cold DPBS, the tubes were kept on ice with light blocking until analysis. The cells were analyzed using the 488 nm of a BD flow cytometer (BD FACSCalibur^TM^ and BD Acuuri^TM^ C6 Plus Flow Cytometer) for excitation, and the data were collected using the FITC-A detector. A minimum of 1 × 10^4^ cells was analyzed for each condition. The BD Accuri C6 Plus software was used for data analysis.

### RNAi-mediated gene knockdown

The cells were seeded at 2 × 10^5^/well in a six-well cell culture plate. ON-TARGET plus SMARTpool for human NFE2L2 siRNA (L-003755-00-0005) and ON-TARGET plus a non-targeting pool (D-001810-20) were purchased from Dharmacon (USA). Lipofectamine RNAiMax (Thermo, 13778150; Invitrogen) was used to transfect siRNA into cells at a final concentration of 20 nM according to the manufacturer’s protocol.

### Total RNA extraction and RT-qPCR

TRIzol^TM^ Reagent (Thermo, 15596018; Invitrogen) was used to extract total RNA according to the manufacturer’s protocol. To synthesize complementary DNA (cDNA), 2 μg of total RNA were used using M-MLV reverse transcriptase (Promega, M1701) according to the manufacturer’s protocol. cDNA (24 ng) was then mixed with 2X Real-Time PCR Smart mix with Evagreen fluorescent dye (Solgent, SRH71-M40H) and analyzed using the CFX96^TM^ Real-Time System (Bio-Rad Laboratories).

The primer sequences were as follows: NRF2 (forward), 5′-ACACGGTCCACAGCTCATC-3′; NRF2 (reverse), 5′-TGTCAATCAAATCCATGTCCTG-3′; KEAP1 (forward), 5′-ATTGGCTGTGTGTGGAGTTGC-3′; KEAP1 (reverse), 5′-CAGGTTGAACTCCTCTTG-3′; NQO1 (forward), 5′- CGTTTCTTCCATCCTTCCAG-3′; NQO1 (reverse), 5′-CGCAGACCTTGTGATATTCC-3′; Ferritin heavy chain (FTH)-1 (forward), 5′-GTTGTACCAAAACATCCACTTAAG-3′; FTH-1 (reverse), 5′-CCTCAAAGACAACACCTGGG-3′; FSP1 (forward), 5′-TCCGTCCGGCAGGAAGTGAA-3′; FSP1 (reverse), 5′-CATTGAGAGGCAGCTCCTCC-3′; GPX4 (forward), 5′-GCTGTGGAAGTGGATGAAGA-3′; GPX4 (reverse), 5′-CAGCCGTTCTTGTCGATGA-3′; β-Actin (forward), 5′-CTGGCACCCAGCACAATG-3′; β-Actin (reverse), 5′-GCCGATCCACACGGAGTACT-3′. The NQO1 sequence was referenced by Bae et al. [34]. All primers, except for GPX4, were purchased from Cosmo Genetech (Korea). GPX4 primers were purchased from Integrated DNA Technologies (USA).

### Western blotting

For western blotting, the cells were lysed in a lysis buffer (50 mM Tris-HCl (pH 7.5), 150 mM NaCl, 0.5% NP-40, 0.5% Triton X-100, 0.1% Na-deoxycholate, and 1 mM EDTA containing a protease inhibitor cocktail). Nitrocellulose membranes were used for the experiment, and after 1 h blocking in 5% skim milk, the primary antibody was incubated at 4 °C overnight. The secondary antibody was then incubated for 1 h at room temperature on a rocker. Western blot images were analyzed using FUSION Solo (Vilber Lourmat) and Evolution Capt software. The antibodies used are as follows: anti-NRF2 (Proteintech, 16396-1-AP), anti-KEAP1 (Proteintech, 10503-2-AP), anti-GPX4 (Abcam, ab125066), anti-FSP1 (Santa cruz, sc-377120), anti-HSP90 (Santa cruz, sc-13119), anti-β-actin (Sigma-Aldrich, A5316), and anti-α-tubulin (Cell signaling, 3873 S).

### Statistical analyses

All experiments were performed at least three times. The data are presented as mean ± standard deviation (SD). Significant differences between the two groups were measured using Student’s t-test and two-way analysis of variance (ANOVA). Statistical analyses were performed using R (v4.2.1), GraphPad Prism 9 (GraphPad Software), and Excel 2016 (Microsoft). Significance was set at *p* < 0.05 (*), *p* < 0.005 (**), and *p* < 0.0005 (***).

## Results

### KEAP1 mutations and consequent NRF2 activation are prominent in ferroptosis-resistant NSCLC

**Fig. 1:**
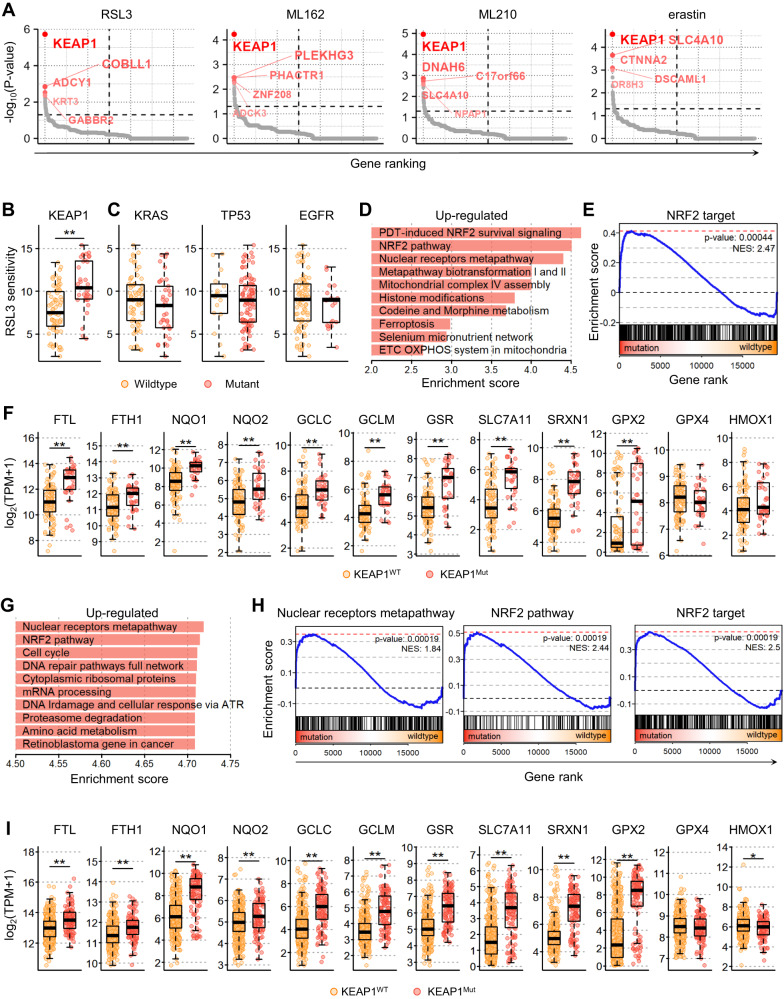
NRF2 activation following *KEAP1* mutations led to ferroptosis resistance in NSCLC. **A**–**F** Observations in the NSCLC cell line datasets obtained from the DepMap portal. **A** Gene raking based on the significance of mutation enrichment in cell lines resistant to the indicated FINs. **B**, **C** RSL3 drug sensitivity of cell lines grouped by the mutation status of *KEAP1* (**B**) and KRAS, TP53, and EGFR (**C**), with each data point representing a single cell line and lower AUC values indicating higher sensitivity to FIN treatment. **D** The top ten significantly enriched pathways in *KEAP1* mutant cells compared to wild-type cells identified by performing GSEA. **E** GSEA plots showing the enrichment of NRF2 targets in gene rank based on differential expression between *KEAP1* mutant vs. wild-type cells. **F** mRNA expression levels (log2TPM + 1) of key antioxidant response element (ARE) genes in cell lines grouped by *KEAP1* mutation status. WT, wild type; Mut, mutant. **G**–**I** Observations from patient samples in the TCGA LUAD cohort obtained from the GDC data portal. **G** The top ten significantly enriched pathways in *KEAP*1 mutant samples compared to wild-type samples by performing GSEA. **H** GSEA plots displaying the enrichment of genes involved in the nuclear receptors metapathway, NRF2 pathway, and NRF2 targets in *KEAP1* mutant vs. wild-type samples. **I** mRNA expression levels of key ARE genes in samples grouped by *KEAP1* mutational status.

To identify key molecules involved in ferroptosis resistance in cancer, we leveraged genomic or transcriptomic data of a thousand human cancer cell lines and their pharmacological responses against FINs, including RSL3, ML210, and ML162, which targeting GPX4, and erastin, which is a system x_c_^-^ inhibitor, obtained from the DepMap database. We first investigated the genetic alterations closely associated with cellular responses to FINs in NSCLC. Among the 16,616 genes with mutations in various cancers, we found that *KEAP1* mutations were the most significantly enriched in NSCLC cell lines that exhibited resistance to FINs (Fisher’s exact test, p < 1e-04; Figs. 1A and S1A). NSCLC cell lines with wild-type *KEAP1* were more susceptible to FINs than those with *KEAP1* mutations (*p* = 8.1e-07, Fig. 1B). In contrast, although RSL3 and erastin were initially identified as cancer drugs selective for tumor cells bearing oncogenic RAS [35, 36], cellular responses to either drug were not associated with the mutational status of *KRAS* (Figs. 1C and S1A) [35]. In addition, *TP53* or *EGFR* play a crucial role in ferroptosis in some contexts [37–40], but mutations in them did not appear to affect cell line sensitivity to FINs (Figs. 1C and S1A). The *KEAP1* gene is a tumor suppressor gene that is frequently mutated in lung adenocarcinoma patients, with a prevalence rate of 17% [41]. *KEAP1* mutations were predominantly identified in NSCLC cell lines (Fig. S1B) and patients (Fig. S1C), and have also been detected in several other cancer types, including liver, ovarian, and uterine cancers (Fig. S1B, C). Notably, cell lines harboring *KEAP1* mutations were generally resistant to FINs in other cancer types (Fig. S1D). However, this resistance was more pronounced in NSCLC (Fig. S1E). These results imply that *KEAP1* mutations may confer ferroptosis resistance in cancers, particularly NSCLC.

To further understand the downstream mechanisms by which *KEAP1* mutations affect ferroptosis resistance in NSCLC, we investigated the altered signaling pathways in *KEAP1*-mutated NSCLC cells compared to *KEAP1* wild-type cells. GSEA using the WikiPathways database [12] revealed that genes involved in the NRF2 signaling pathway were significantly upregulated in *KEAP1*-mutated NSCLC cells compared to NSCLC cells with wild-type *KEAP1* (Fig. 1D). This result is consistent with the well-established role of *KEAP1* mutation in activating the NRF2 pathway [22], as seen in the increased expression levels of most NRF2 target genes in *KEAP1*-mutated NSCLC cells (Fig. 1E). *KEAP1* mutant cells exhibited significantly higher expression levels of NRF2 target genes, that are involved in iron homeostasis, GSH metabolism, or ROS detoxification, including *FTH1*, ferritin light chain (*FTL*), NAD(P)H quinone dehydrogenase 1 (*NQO1*), glutamate-cysteine ligase catalytic subunit (*GCLC*), glutamate-cysteine ligase regulatory subunit (*GCLM*), solute carrier family 7 member 11 (*SLC7A11*), glutathione-disulfide reductase (*GSR*), sulfiredoxin 1 (*SRXN1*), and glutathione peroxidase 2 (*GPX2*) (p < 1e-05; Fig. 1F). *GPX4* is thought to be regulated by NRF2, but *KEAP1* mutations did not significantly affect its expression levels, which is supported by a recent study showing that NRF1 regulates *GPX4* independent of NRF2 (Fig. 1F) [42]. There was no significant difference in the expression levels of heme oxygenase-1 (*HMOX1)*, an NRF2 target genes that promotes ferroptosis, in contrast to NRF2’s cytoprotective role (Fig. 1F) [43, 44]. Consistent results were observed in the lung adenocarcinoma (LUAD) patient cohort (Fig. 1G–I), where the NRF2 pathway and nuclear receptor metapathway, which encompass the majority of NRF2 target genes, were significantly upregulated in *KEAP1*-mutated samples (Fig. 1G). The mRNA expression levels of NRF2 targets were significantly elevated in *KEAP1*-mutated LUAD samples compared to those in LUAD samples with wild-type *KEAP1* (Fig. 1I). This significant difference was also observed in the other cancer cohorts (Fig. S1F), implying that *KEAP1* regulates NRF2 activity in various cancers. Overall, our findings suggested that the increased resistance to ferroptosis observed in *KEAP1*-mutated cells may be attributed to the aberrant activation of NRF2.

### Elevated FSP1 expression level is strongly associated with enhanced resistance to ferroptosis in cancer

**Fig. 2:**
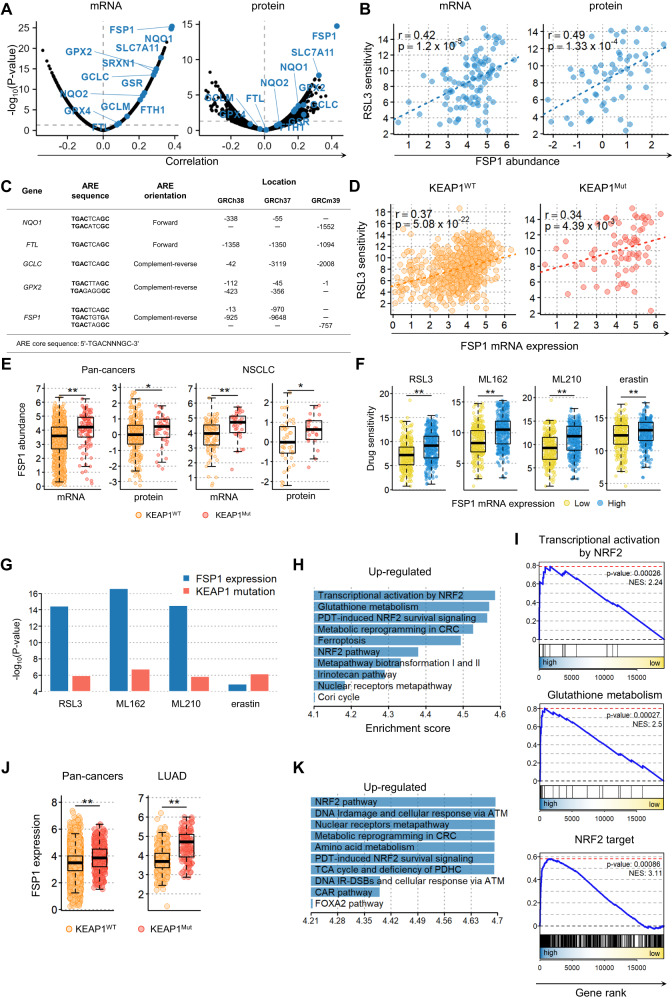
Ferroptosis resistance in cancer is closely associated with increased FSP1 expression level. **A** Gene ranking by Spearman correlation with RSL3 sensitivity based on mRNA expression (left) or protein expression (right) across over 700 cancer cell lines. **B** Correlation between RSL3 sensitivity and FSP1 abundance (mRNA expression, left; protein expression, right) in NSCLC cell lines. **C** Conserved ARE motifs and positions in NRF2 target genes and *FSP1*. Human genome references are GRCh38 and GRCh37, and mouse references are GRCm39. **D** Correlation between RSL3 sensitivity and FSP1 mRNA level in *KEAP1* wild-type (left) and *KEAP1* mutant cells (right) across pan-cancer cell lines. **E** FSP1 mRNA and protein expression levels in pan-cancer and NSCLC cell lines grouped by *KEAP1* mutation status. **F** FIN sensitivity grouped by FSP1 mRNA expression levels. FSP1-high and FSP1-low groups were defined based on the median value of FSP1 expression level across pan-cancer cells. **G** Statistical significance of FIN sensitivity differences in cell lines grouped by *KEAP1* mutation status (red) and FSP1 expression (blue) in pan-cancer cell lines. *p*-value was estimated by the Student’s *t* test. **H** The top ten significantly enriched pathways in FSP1-high cells compared to FSP1-low cells identified by performing GSEA. **I** GSEA plots for gene sets involved in transcriptional activation by NRF2, glutathione metabolism, or NRF2 targets in FSP1-high vs. FSP1-low cells. **J** FSP1 mRNA expression levels in TCGA pan-cancer and LUAD samples grouped by *KEAP1* mutational status. **K** The top ten significantly enriched pathways in FSP1-high compared to FSP1-low TCGA LUAD samples identified by performing GSEA.

To identify the key NRF2 targets contributing to ferroptosis resistance, we performed a comprehensive search for genes whose expression was highly associated with cell line sensitivity to FINs. We assessed 19,221 genes and observed a positive correlation between FIN sensitivity and the expression of several well-known NRF2 targets, including *NQO1, SLC7A11, GPX2, GCLC*, and *FTH1*, across various cancer cell lines at both the mRNA and protein levels (Figs. 2A and S2A, B). Notably, FSP1 exhibited the most significant correlation, which was consistently observed in NSCLC cells (Figs. 2B and S2C). This is noteworthy because FSP1 has previously been identified as a target of NRF2 through ChIP-seq analysis [45] and has recently been shown to be regulated by NRF2 in the context of ferroptosis [46]. Additionally, we detected conserved ARE core sequences located within 1 kb upstream of the transcription start site (TSS) of *FSP1* in both the human (GRCh38, GRCh37) and mouse (GRCm39) reference genomes. These motifs were found on the opposite strand of the coding strand in a reverse complementary orientation, similar to those in the *GPX2* gene, suggesting that NRF2 regulates the transcription of *FSP1* (Fig. 2C) [46, 47]. Consistent with this observation, FSP1 expression level was higher in *KEAP1* mutant cells than in wild-type cells, similar to other NRF2 targets (Fig. 2D), and maintained a significant correlation with RSL3 sensitivity in both *KEAP1* mutant and wild-type cells (Fig. 2E). This implies that FSP1 abundance could serve as a more reliable predictor of ferroptosis resistance than the mutational status of *KEAP1*. Indeed, the difference in FIN sensitivity between cells grouped by FSP1 expression was more significant than that between cells grouped by *KEAP1* mutation status (Fig. 2F, G). GSEA between FSP1-high and FSP1-low cell groups revealed a significant upregulation of numerous NRF2-related gene signatures, including transcriptional activation by NRF2, GSH metabolism, and NRF2 targets in cells with higher FSP1 expression levels (Fig. 2H, I). Consistent with the cell line data, a significant increase in FSP1 abundance was observed in individuals with KEAP1 mutations across both the pan-cancer and LUAD cohorts (Fig. 2J). Significant upregulation of NRF2-related pathways was observed in individuals with high FSP1 expression levels (Fig. 2K). Our findings from both cell line and patient datasets suggest that FSP1 plays a key role in mediating ferroptosis resistance related to NRF2, highlighting the potential of FSP1 as a promising therapeutic target for the treatment of ferroptosis-resistant cancers.

### NRF2 activity and FSP1 expression are critical determinants for ferroptosis resistance

**Fig. 3:**
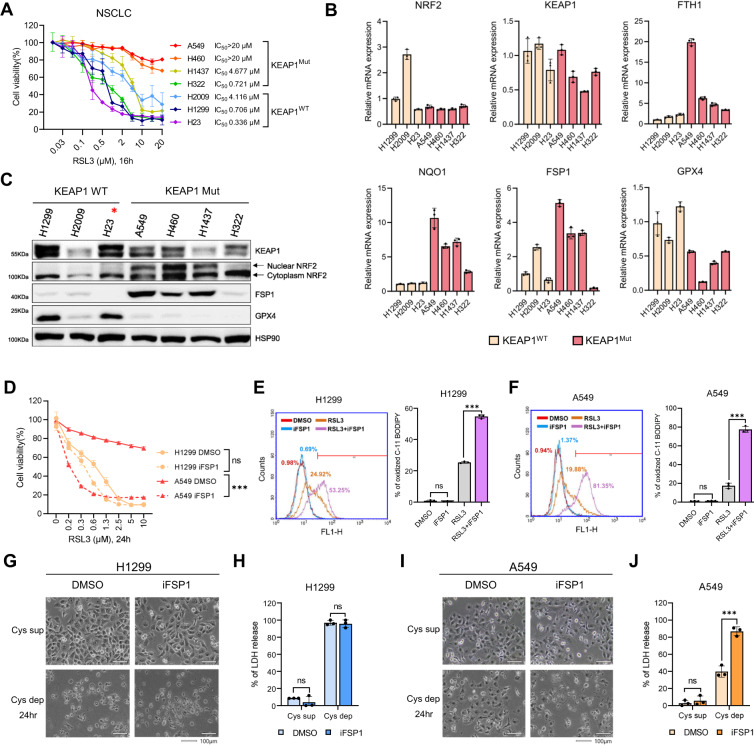
FSP1 inhibition increases the sensitivity of KEAP1 mutant NSCLC to ferroptosis. **A** Relative cell viability of NSCLCs treated with an increasing concentration of RSL3 (μM) for 16 h. IC_50_ was calculated by GraphPad Prism 9 software. **B** The relative mRNA expression levels of ferroptosis-related genes in *KEAP1* wild-type and mutant NSCLC were measured by qRT-PCR. All mRNA expression levels were normalized to *β* –Actin mRNA expression levels. **C** Western blot analysis in NSCLC was classified according to *KEAP1* mutation status. The red star marks that H23 cells were exceptionally classified as *KEAP1* wild-type because they harbor a mutation that does not affect KEAP1 protein function. **D** Relative cell viability of NSCLCs treated with the various concentrations of RSL3 ranging from 0 μM to 10 μM for 24 h. Cells were co-treated with RSL3 and DMSO or 3 μM of iFSP1. (E and F) Lipid peroxidation assessment in H1299 (**E**) and A549 (**F**) cells at 45 min after RSL3 (1 μM) or/and iFSP1 (3 μM) treatments using C-11 BODIPY (2.5 μM). The results are summarized as a bar graph showing relative levels in the right panel. **G** Microscopic morphology of H1299 cultured in cystine-deficient medium in the presence of iFSP1 (3 μM) for 24 h. The white line represents a 100 μm scale bar. **H** Relatively released LDH levels of H1299 cells treated with iFSP1 (3 μM) in cystine-deficient medium for 24 h. **I**, **J** Morphology and released LDH levels of A549 cells treated and measured as in **G**, **H**. All data are presented as the mean ± S.D (*n* = 3 independent experiments). The *p*-value is significant for *p* < 0.05 and the measurement of the value was according to the Student’s *t* test and one-way ANOVA.

For further investigation, we used seven NSCLC cell lines, including four that harbor *KEAP1* mutations (A549, H460, H1437, and H322) and three that harbor wild-type *KEAP1* (H1299, H2092, and H23). We examined the sensitivity of these cell lines to RSL3. Cell lines without *KEAP1* mutations demonstrated a dose-dependent decrease in viability upon RSL3 treatment, whereas those with *KEAP1* mutations showed overall resistance to RSL3 (Fig. 3A). These results suggested that *KEAP1* mutations confer resistance to ferroptosis. Upon assessment of mRNA expression via real-time PCR, there was no significant difference in *NRF2* and *KEAP1* levels between *KEAP1* wild-type and mutant cells (Fig. 3B). However, the mRNA expression levels of NRF2 target genes, such as *NQO1*, *FTH1*, and *FSP1*, were significantly higher in the *KEAP1* mutant cell lines (Fig. 3B). In contrast, the mRNA expression level of GPX4 was rather higher in cells with wild-type *KEAP1* (Fig. 3B) [42]. Consistent with the mRNA expression pattern, the protein levels of FSP1 and GPX4 showed a similar pattern in *KEAP1* wild-type and mutant cells (Fig. 3C). H23 cells (denoted by a red star at Fig. 3C) possessed the *KEAP1*-Q193H mutation; however, we classified them as *KEAP1* wild-type because this mutation does not cause functional KEAP1 abnormalities. H23 cells indeed exhibit lower levels of nuclear NRF2 protein and NRF2 target genes, which were comparable to those observed in other *KEAP1* wild-type cells (Fig. 3B, C) [48]. Notably, H322 cells with *KEAP1* mutations showed lower expression levels of NRF2 target genes and decreased FSP1 and GPX4 protein expression levels. Western blot analysis revealed that the nuclear form of NRF2 was significantly reduced in H322 cells, whereas cytoplasmic NRF2 levels remained high (Fig. 3C). The detailed mechanism of this phenomenon is yet to be determined, as no mutations in NRF2 were identified in H322 cells, but it is apparent that impaired NRF2 nuclear translocation resulted in reduced expression levels of NRF2 target genes, including *FSP1*, rendering the cells more susceptible to RSL3 (Fig. 3A). Overall, these findings suggest that the presence of *KEAP1* mutations alone may not be sufficient to indicate ferroptosis resistance. NRF2 activity and FSP1 expression, rather than *KEAP1* status, appeared to be more critical determinants of ferroptosis sensitivity in NSCLC.

### Inhibition of FSP1 sensitizes KEAP1 mutant cells to ferroptosis

We further evaluated the effects of FSP1 on ferroptosis sensitivity using iFSP1 in two representative NSCLC cell lines, H1299 (*KEAP1* wild-type) and A549 (*KEAP1* mutant) (Fig. 3D) [13, 14]. H1299 cells expressing low levels of FSP1 were sensitive to ferroptosis induced by RSL3, a GPX4 inhibitor. However, treatment with iFSP1 only slightly increased their sensitivity to RSL3 (Fig. 3D). In contrast, A549 cells with high FSP1 expression level and RSL3 resistance were sensitized to RSL3 in the presence of iFSP1 (Fig. 3D). Lipid peroxidation was elevated in H1299 cells treated with both RSL3 and iFSP1, suggesting that FSP1 inhibition may facilitate lipid peroxidation (Fig. 3E). Additionally, lipid peroxidation levels in A549 cells were extremely low after RSL3 treatment, but populations of cells with high levels of lipid peroxidation after RSL3 treatment increased more than fourfold in iFSP1-treated cells compared to DMSO-treated cells (Fig. 3F). Furthermore, iFSP1 treatment alone did not induce lipid peroxidation in either H1299 or A549 cells, implying that GPX4 may be sufficient to remove lipid peroxides even in the absence of FSP1 (Figs. 3E, F). However, once lipid peroxides accumulate when GPX4 is inactivated, FSP1 may play a pivotal role in suppressing ferroptosis. As a result, inhibition of FSP1 may be beneficial for cancer therapy targeting ferroptosis, particularly in resistant cancer cells with high FSP1 expression levels.

To ascertain whether the sensitizing effect of iFSP1 was dependent on FSP1 expression or *KEAP1* status, we determined the sensitivity of other NSCLC cell lines to RSL3 in the absence or presence of iFSP1. In H2009 and H23 cells, which are *KEAP1* wild-type and exhibited low FSP1 expression, no significant synergistic effect of iFSP1 treatment on RSL3-induced ferroptosis was observed (Fig. S3A). In addition, H460 and H1437, which are *KEAP1* mutant that expressed high levels of FSP1 protein, were greatly sensitized to ferroptosis upon iFSP1 treatment (Fig. S3B). Interestingly, iFSP1 also sensitized H322 cells, an FSP1-low cell line with low nuclear NRF2 levels despite *KEAP1* mutations, to ferroptosis (Fig. S3B). This result indicates that although FSP1 levels were low in H322 cells, their expression was sufficient to inhibit ferroptosis, suggesting that the activity of FSP1 beyond its expression may be more important for ferroptosis sensitivity. Considering *KEAP1* mutation in H322 cells, KEAP1 may be involved in the quality control of the FSP1 protein, which requires further investigation.

Next, we investigated whether FSP1 plays a crucial role in ferroptosis caused by cysteine depletion in *KEAP1* mutant NSCLC. The depletion of cysteine consequently reduces intracellular GSH levels and induces ferroptosis by lowering GPX4 activity in vitro and in vivo [1, 49]. When cysteine was depleted from the medium after 24 h, H1299 cells showed necrotic morphology in the presence and absence of iFSP1 (Fig. 3G). LDH release analysis revealed that almost all H1299 cells died after cysteine deprivation, regardless of iFSP1 treatment (Fig. 3H). In contrast, A549 cells exhibited less necrotic morphology after cysteine deprivation, but massive cell death was observed in A549 cells treated with iFSP1 in the cysteine-depleted medium (Figs. 3I, J). Under the preceding conditions, cell death was inhibited by Fer-1 treatment, and A549 cells survived in the absence of iFSP1 in Cys-depleted medium even after 48 h (Fig. S3C, D). In summary, FSP1 is a key driver of ferroptosis resistance in *KEAP1* mutant NSCLC cells. As a result, simultaneous inhibition of FSP1 and GPX4 effectively kills *KEAP1*-mutant NSCLC cells.

### NRF2 depletion is insufficient to deplete FSP1 protein but enhances ferroptosis in the presence of RSL3 and iFSP1

**Fig. 4:**
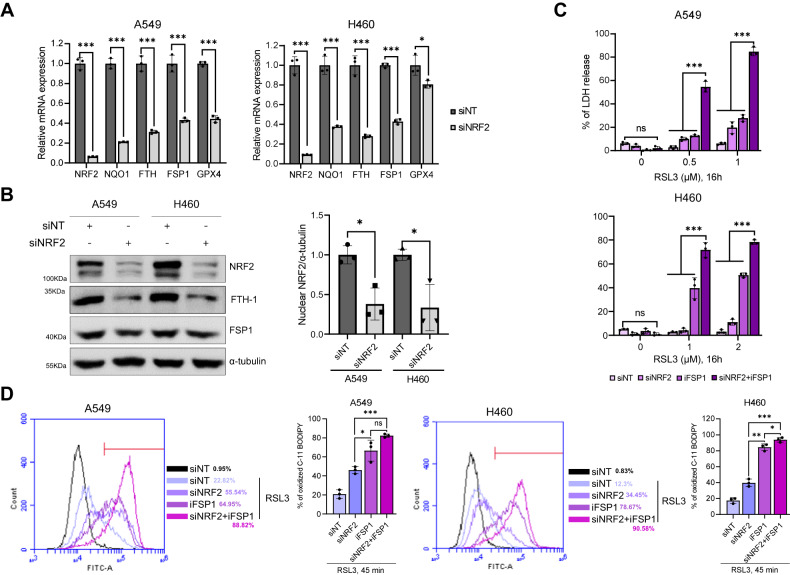
FSP1 inhibition renders cells more susceptible to ferroptosis than NRF2 downregulation. **A** Relative mRNA expression of NRF2 target genes in A549 and H460 upon NRF2 knockdown. Cells were transfected with a non-targeting siRNA pool (siNT) or an NRF2 siRNA pool (siNRF2) for 48 h, followed by RT-PCR analysis. All mRNA expression levels were normalized to *β*-Actin mRNA expression levels. **B** Western blot analysis showing NRF2, FTH-1, and FSP1 in A549 and H460 upon NRF2 knockdown for 48 h as in **A**. Representative western blots are shown in the left panel, and three independent analyses are shown as a bar graph in the right panel. Each target protein was quantified by α-tubulin. **C** Cell death measured by the LDH release of A549 and H460 cells transfected with siNRF2 for 48 h, followed by the treatment with RSL3 (0.5 and 1 μM) and iFSP1 (3 μM) for 16 h. **D** A histogram showing lipid peroxidation levels of control- and NRF2-depleted A549 and H460 cells treated with RSL3 (1 μM) and iFSP1 (3 μM) for 45 min. A bar graph quantifying lipid peroxidation was presented in the right panel. Analysis was performed 15 min after C-11 BODIPY (2.5 μM) treatment. All data are presented as the mean ± S.D (*n* = 3 independent experiments). The *p*-value is significant for *p* < 0.05 and the measurement of the value was according to Student’s *t* test.

Given that NRF2 is a transcription factor for FSP1 [46], we examined whether inhibiting NRF2 would have the same effect as targeting FSP1 directly in enhancing ferroptosis in NSCLC with *KEAP1* mutation. Depletion of NRF2 successfully diminished the expression levels of NRF2 target genes, such as *NQO1, FTH, and FSP1*, as was also evident from two independent microarray datasets (Figs. 4A and S4A) [50, 51]. Interestingly, western blotting analysis showed that NRF2 and FTH-1 protein levels were significantly reduced upon NRF2 knockdown, whereas GPX4 protein expression was increased upon NRF2 knockdown despite a decrease in GPX4 mRNA expression, suggesting the possible involvement of NRF2 in the translation and incorporation of selenocysteine, a major mechanism for regulating GPX4 protein expression (Fig. 4B and Fig. S4B). However, there was only a slight reduction in FSP1 protein expression levels despite a more than 2-fold decrease in *FSP1* expression level (Fig. 4B). These data imply that FSP1 is not solely transcriptionally controlled by NRF2; therefore, inhibiting NRF2 may not be sufficient to deplete the FSP1 protein. NRF2 knockdown did not facilitate RSL3-induced ferroptosis, but significantly increased cell death upon treatment with RSL3 and iFSP1 for 16 h (Fig. 4C). iFSP1 slightly induced ferroptosis in A549 cells treated with RSL3 for 16 h, but it drastically induced cell death after 24 h of stimulation (Figs. 4C and S4C). Compared to H460 cells, in which iFSP1 was effective in increasing RSL3-induced ferroptosis, A549 cells exhibited stronger NRF2 activity, which may attenuate ferroptosis when both GPX4 and FSP1 are inhibited (Figs. 3B, 4C, and S4C). NRF2 depletion slightly increased early lipid peroxidation in A549 and H460 cells treated with RSL3 for 45 min, but it had no significant effect on lipid peroxidation in cells treated with RSL3 and iFSP1 (Fig. 4D). These data suggest that even when NRF2 is depleted, residual levels of FSP1 may be sufficient to scavenge lipid peroxyl radicals and protect cells from ferroptosis. NRF2 protects against ferroptosis when both GPX4 and FSP1 are inactivated in *KEAP1* mutant NSCLC cells. Overall, in *KEAP1* mutant NSCLC, NRF2 does not fully control the FSP1, indicating that inhibiting NRF2 alone does not overcome RSL3-induced ferroptosis resistance. Furthermore, NRF2 target genes confer resistance to early ferroptosis independent of FSP1, implying that dual targeting of NRF2 and FSP1 is an effective strategy for inducing ferroptosis.

## Discussion

NRF2 is a well-characterized transcription factor that regulates antioxidant and detoxifying genes to maintain cellular redox homeostasis in response to oxidative stress [52, 53]. Additionally, NRF2 exerts a protective role against ferroptotic stimuli [32, 54]. Each NRF2 target gene plays a critical role, but it is unclear which is the most important or whether all genes are required for the anti-ferroptotic activity of NRF2, owing to a lack of research on the impact of individual target genes on ferroptosis. In this study, we found that the *KEAP1* mutation is a strong indicator of ferroptosis resistance in NSCLC and is accompanied by the activation of various NRF2 target genes, such as *FSP1*, *NQO1*, *SLC7A11*, *GPX2*, *SRXN1*, and *GCLC*, by investigating omics datasets obtained from over 1,000 cancer cell lines and hundreds of primary cancer tissues. The expression of several NRF2 target genes positively correlated with low ferroptosis sensitivity, but *FSP1* was found to be the most significant gene associated with ferroptosis resistance. FSP1 was identified as a crucial protein that strongly inhibits ferroptosis, and its expression is known to be regulated by NRF2 [13, 14, 46].

In this study, we attempted to elucidate whether the anti-ferroptotic activity of NRF2 is primarily mediated by FSP1 or by classical NRF2 target genes. Notably, NRF2 downregulation failed to sensitize cells to RSL3-induced ferroptosis in A549 and H460 cells, whereas FSP1 inhibition significantly facilitated ferroptosis. As previously reported, we also observed that knockdown of NRF2 substantially reduced the mRNA levels of FSP1; however, it failed to reduce the abundance of FSP1 proteins. We cannot exclude the possibility that residual NRF2 expression or activity maintains specific levels of NRF2 target genes, including FSP1, resulting in an insufficient promotion of ferroptosis. Additionally, FSP1 protein may still be present owing to its slow turnover, and that posttranslational modifications and protein–protein interactions contribute to the stabilization of FSP1 protein. Several recent studies have suggested that FSP1 expression is affected by other transcription factors, such as activation of transcription factor 6 (ATF6) and the farnesoid X receptor (FXR), suggesting the NRF2-independent regulation of FSP1 [55, 56]. Nevertheless, NRF2 depletion can intensify ferroptosis when GPX4 and FSP1 are inhibited, suggesting an FSP1-independent role for NRF2.

As a result, we suggest that NRF2 and FSP1 suppress ferroptosis via distinct pathways. In *KEAP1* mutant NSCLC cells, high activity of FSP1, which might be induced by NRF2 or other factors, can rapidly remove lipid peroxyl radicals when initial lipid peroxides accumulate in cells with GPX4 inhibition, thereby preventing the propagation of lipid peroxidation. Consequently, the inhibition of FSP1, in addition to GPX4, allows for the accumulation of lipid peroxides, leading to ferroptosis upon RSL3 treatment. However, the inhibition or depletion of NRF2 was insufficient to effectively reduce the FSP1 protein level, and the remaining FSP1 was adequate to suppress ferroptosis. The supply of ferritin, GSH, and antioxidant proteins by NRF2 may delay continuous lipid peroxidation and amplification. NRF2 inhibition therefore facilitates lipid peroxidation and ferroptosis in cells treated with GPX4 and FSP1 inhibitors. NRF2 and FSP1 are important regulators of ferroptosis, but simultaneous inhibition of NRF2 and FSP1 was not sufficient to induce lipid peroxidation and ferroptosis (Fig. S4G), indicating that the disruption of the GPX4/GSH system is required for the initiation of ferroptosis.

*KEAP1* mutations are generally associated with ferroptosis resistance due to the activation of NRF2 and FSP1; however, FSP1 expression may also be regulated independently of KEAP1 and NRF2. As a result, NRF2 activity and FSP1 levels, rather than the *KEAP1* genetic status, were clear predictors of ferroptosis resistance. Indeed, FSP1 expression is strongly correlated with ferroptosis sensitivity in a pan-cancer cell line drug sensitivity database. NRF2 inhibition had limited activity toward the reduction of FSP1 and therefore did not have a significant effect on promoting ferroptosis. In conclusion, inhibition of FSP1 with ferroptosis inducers could be a promising therapeutic approach for a wide range of tumors. Furthermore, the concomitant targeting of NRF2 and FSP1 may offer a maximal antitumor effect in *KEAP1*-mutant tumors.

## Supplementary information

Figure S1

Figure S2

Figure S3

Figure S4

Supplementary figure legend

Uncropped werstern blot

## Data Availability

The data generated in this study can be available in the article and supplemental files. Data sets of human cancer cell lines are available on the DepMap portal (https://depmap.org/portal/, version 22Q2). RNA-seq data of human cancer patients are available on the GDC data portal (https://portal.gdc.cancer.gov/). Information on genetic alterations in individual TCGA samples are available on the cBioPortal (https://www.cbioportal.org/). WikiPathways’ gene annotations are available on the MSigDB (https://www.gsea-msigdb.org/gsea/msigdb). Gene expression data of A549 cells with or without NRF2 knock-down are available on the Gene Expression Omnibus (GSE94393 and GSE38332).
