# Supplementary figures and images for: FSP1 confers ferroptosis resistance in KEAP1 mutant non-small cell lung carcinoma in NRF2-dependent and -independent manner
Source: Cell Death Dis. 2023 Aug 26;14(8):567. doi: 10.1038/s41419-023-06070-x (PMC10460413; doi:10.1038/s41419-023-06070-x)

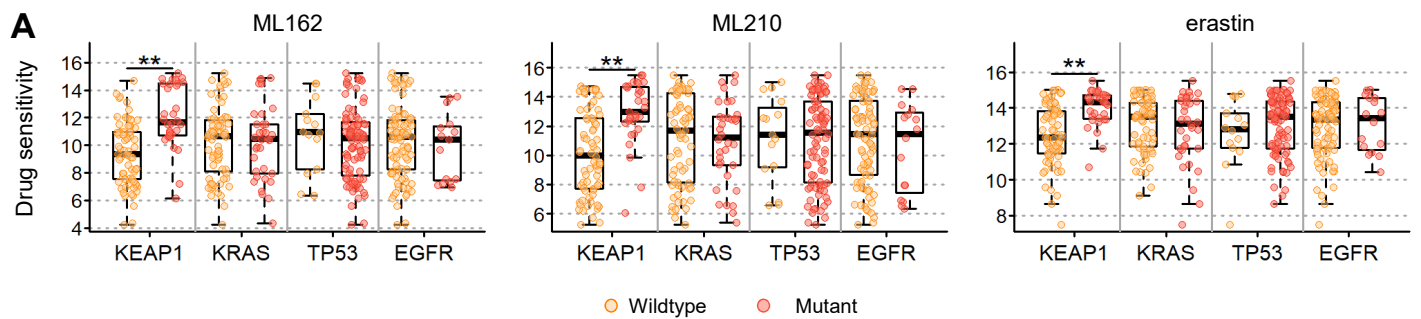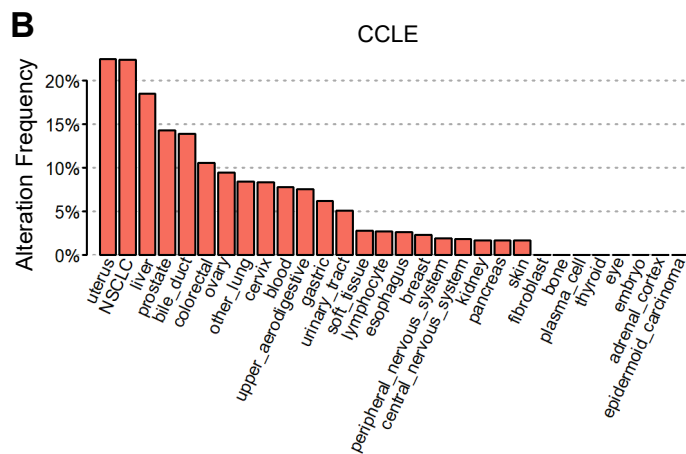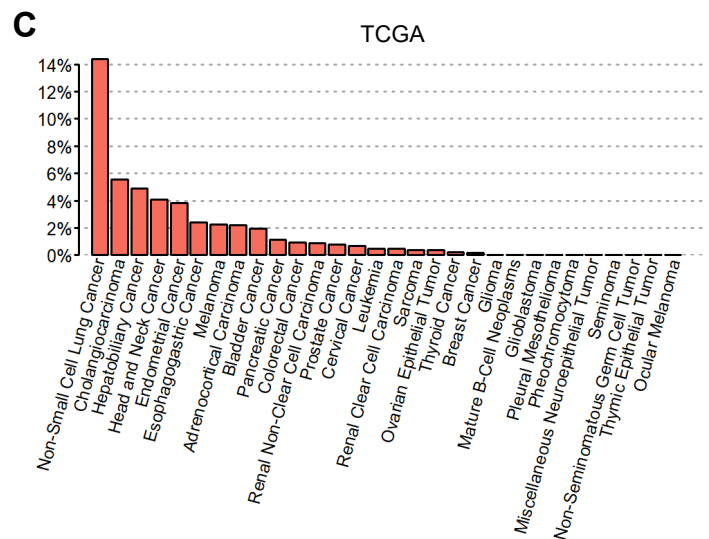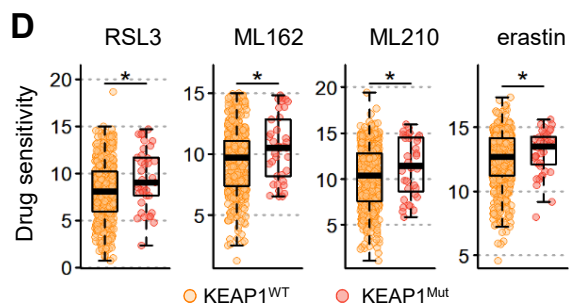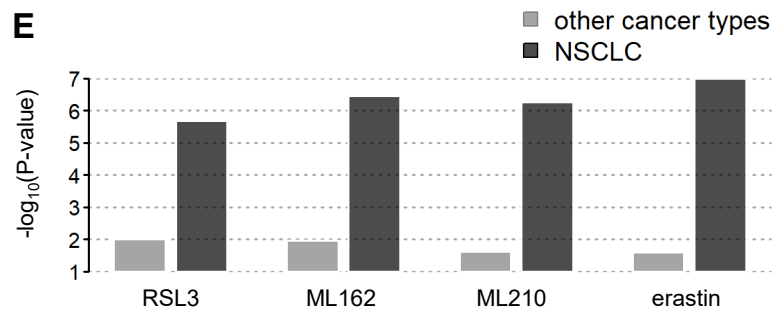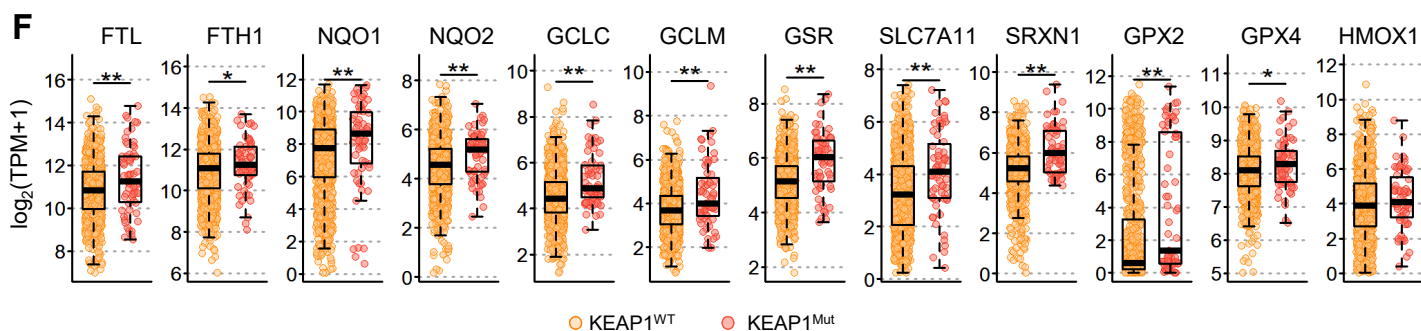

Supplement: Supplementary file 1 — Figure S1 [file 41419_2023_6070_MOESM1_ESM.pdf]

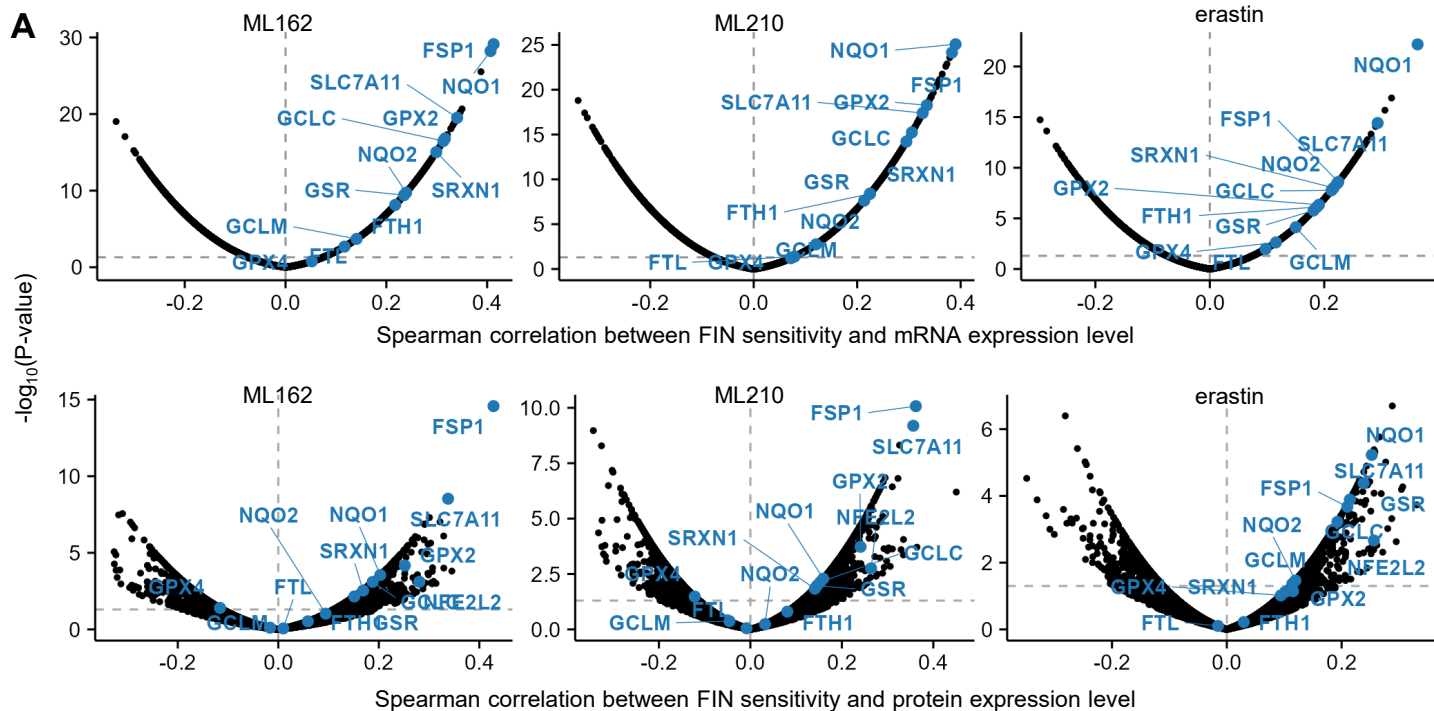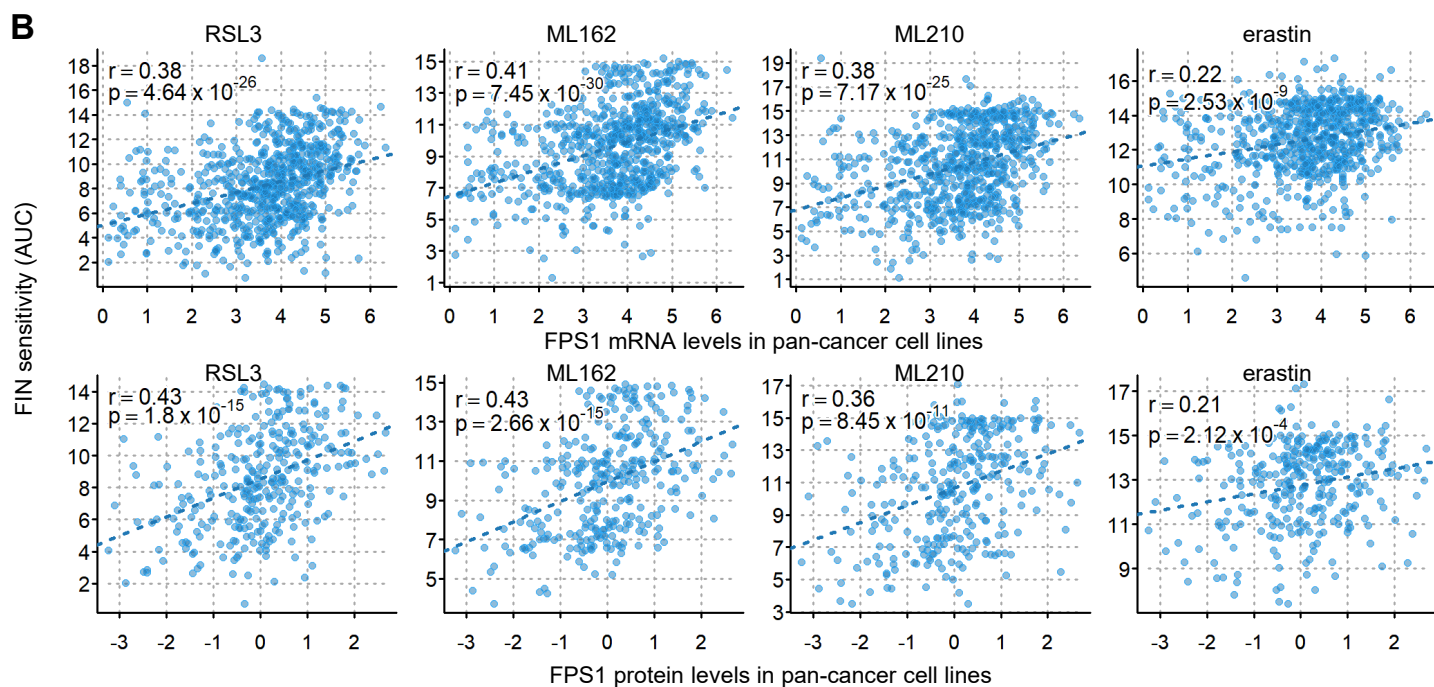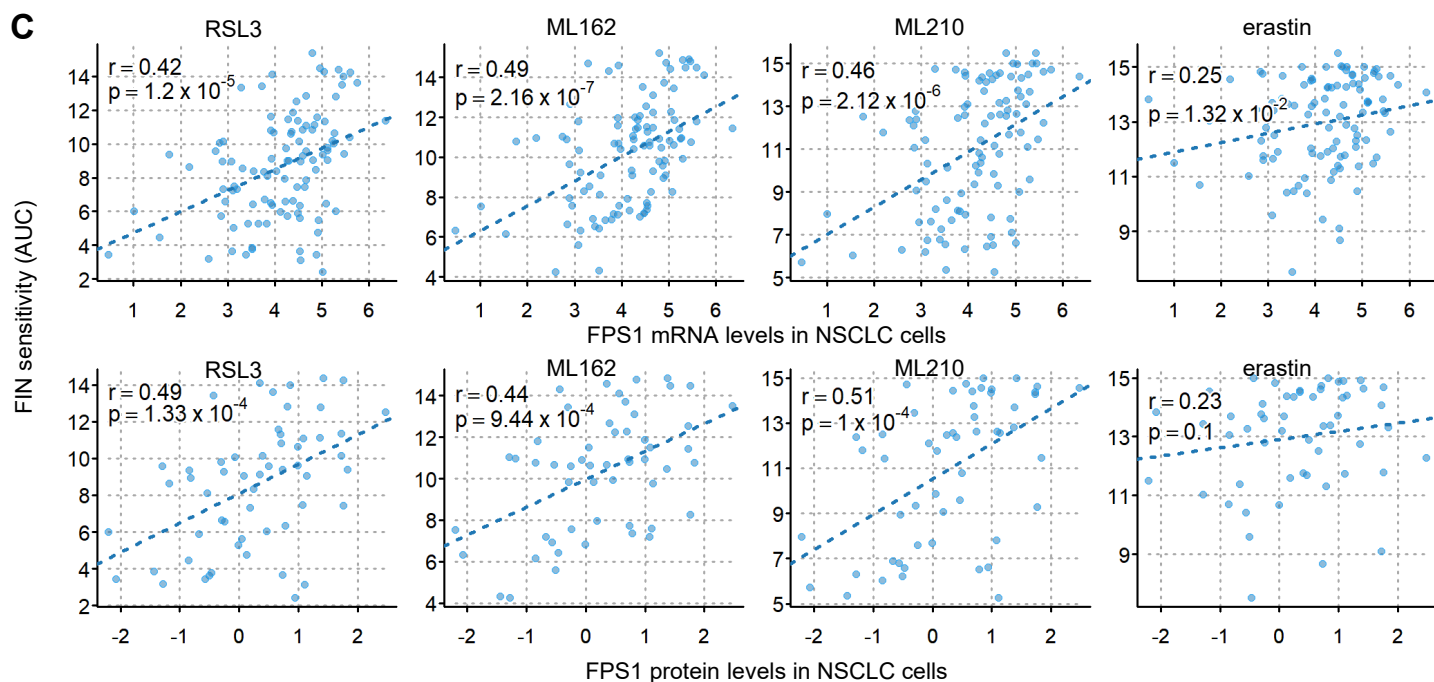

Supplement: Supplementary file 2 — Figure S2 [file 41419_2023_6070_MOESM2_ESM.pdf]

**A**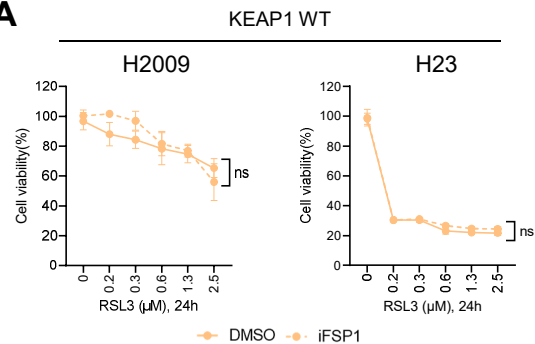**B**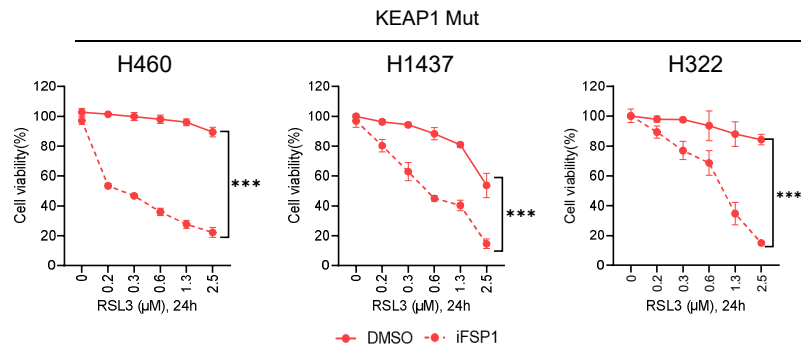**C**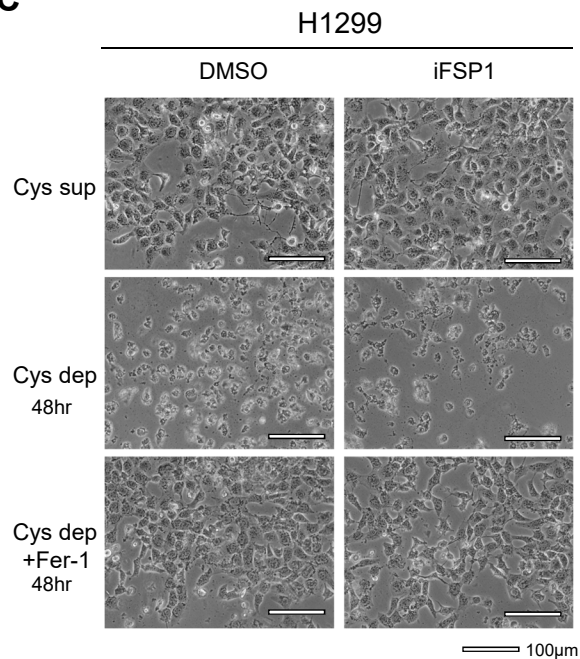**D**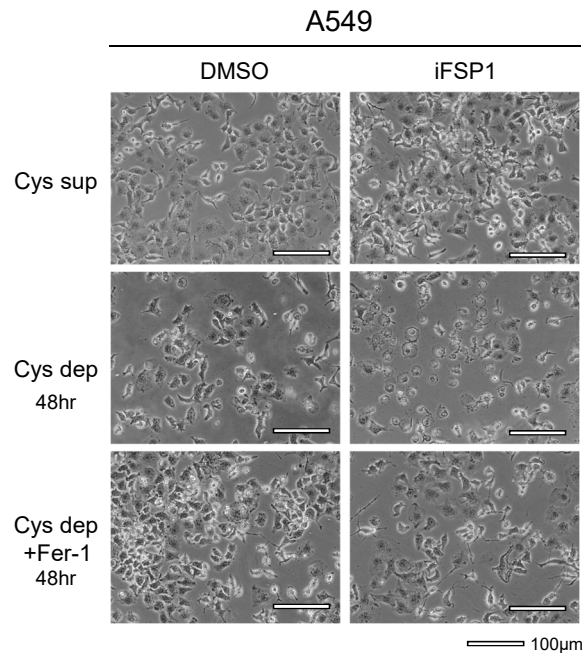

Supplement: Supplementary file 3 — Figure S3 [file 41419_2023_6070_MOESM3_ESM.pdf]

**A**

GSE94393

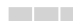**Group**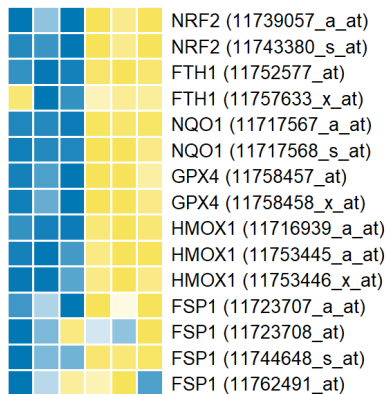

GSE38332

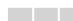**Group**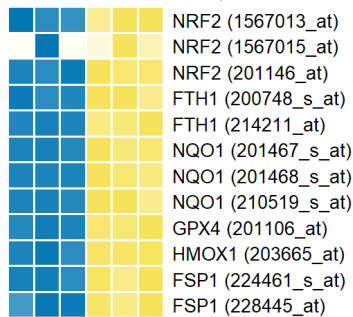

A549 #1  
A549 #2  
A549 #3  
A549 + siRNA #1  
A549 + siRNA #2  
A549 + siRNA #3

Z-score

-1

0

1

**Group**

Control

NRF2 KD

**B**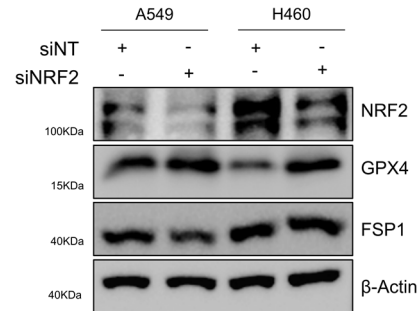**C**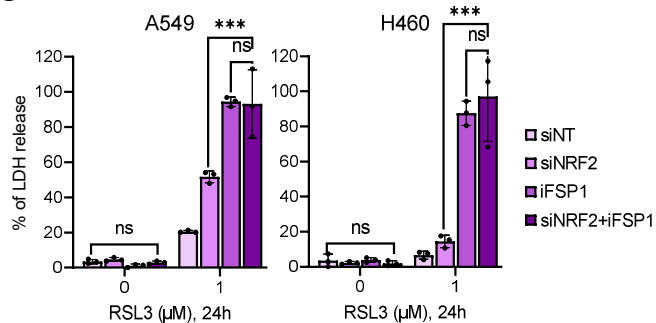

Supplement: Supplementary file 4 — Figure S4 [file 41419_2023_6070_MOESM4_ESM.pdf]

Figure. 3C

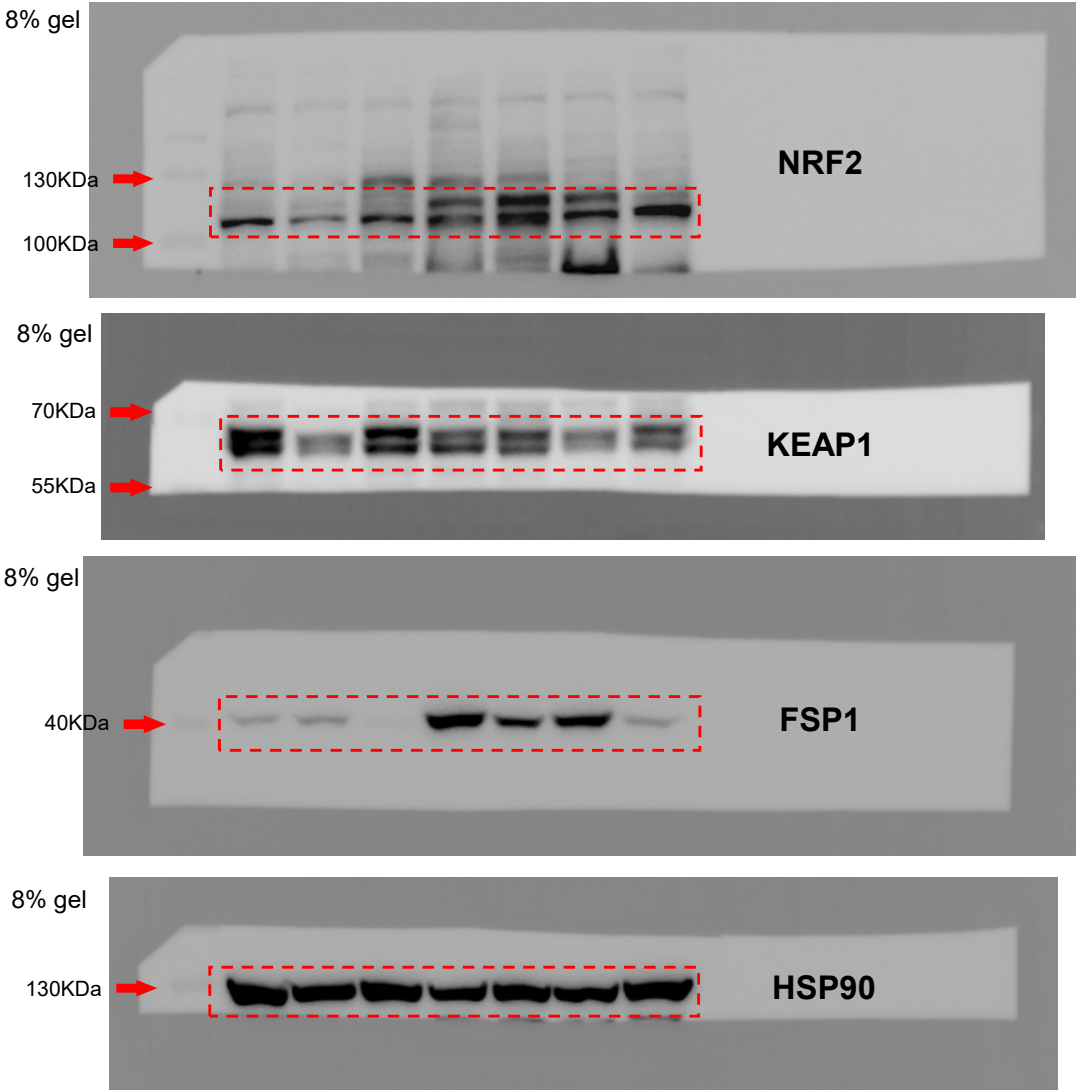

Figure. 4B

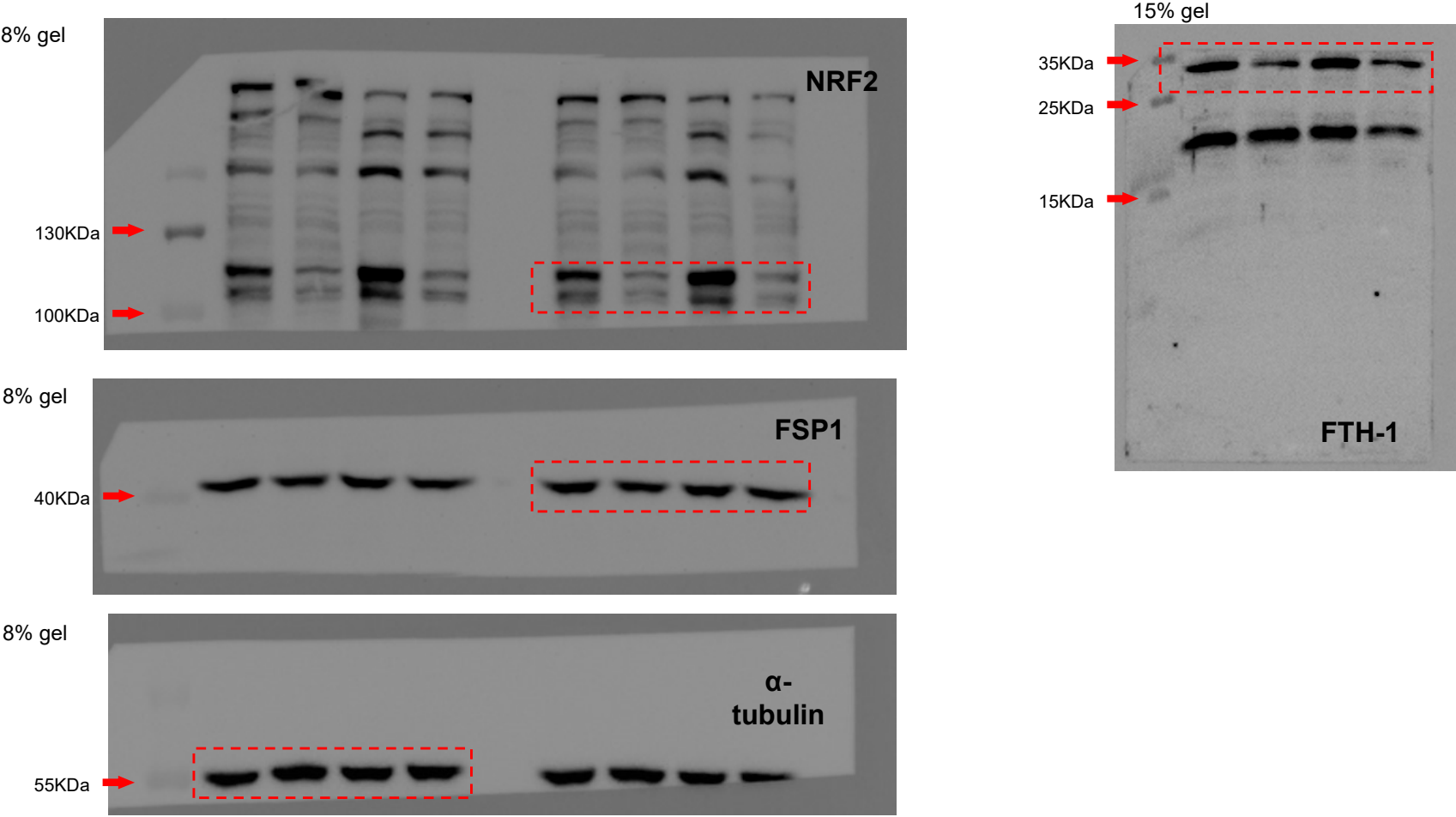

Figure. S4B

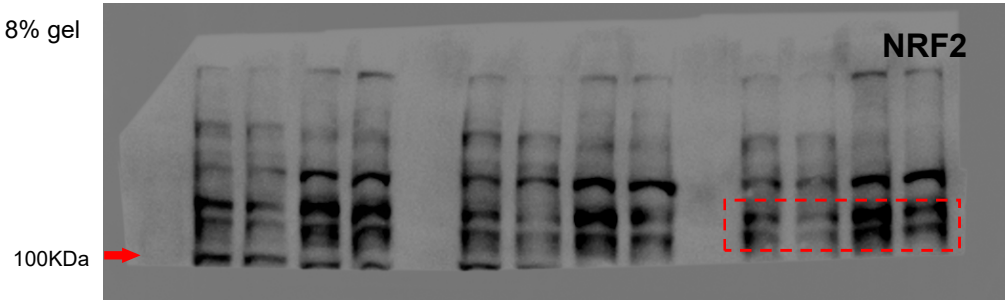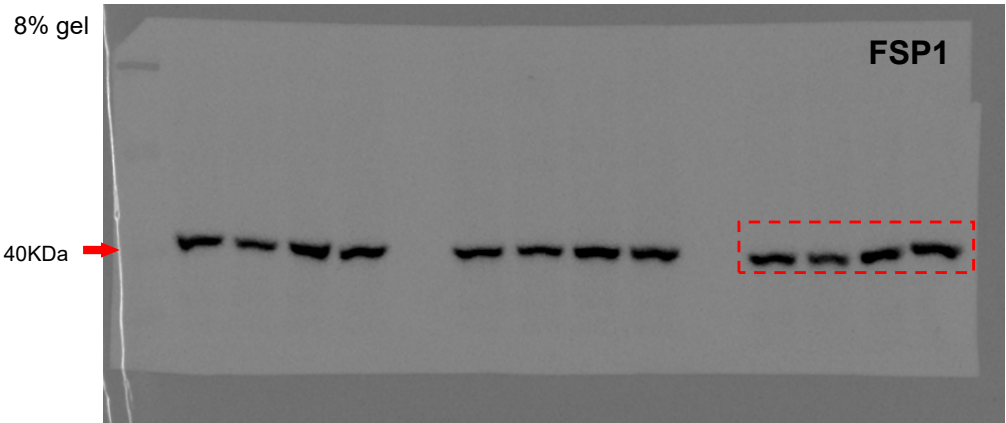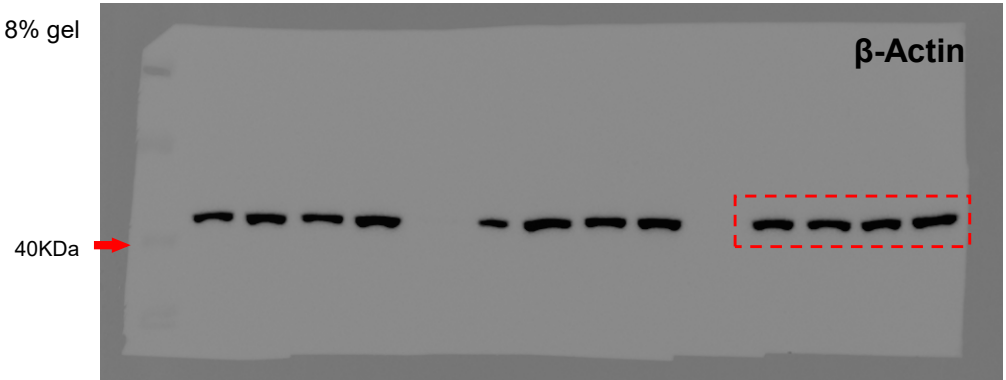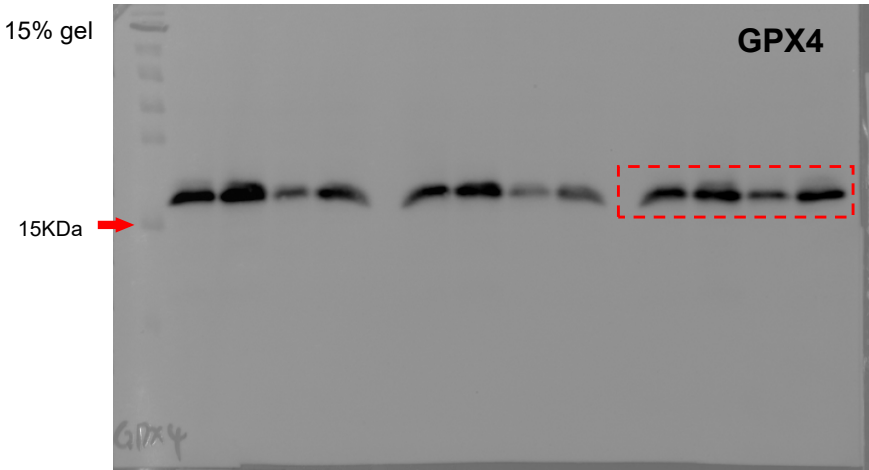

Supplement: Supplementary file 6 — Uncropped werstern blot [file 41419_2023_6070_MOESM6_ESM.pdf]
