# Supplementary material for: FSP1 confers ferroptosis resistance in KEAP1 mutant non-small cell lung carcinoma in NRF2-dependent and -independent manner
Source: Cell Death Dis. 2023 Aug 26;14(8):567. doi: 10.1038/s41419-023-06070-x (PMC10460413; doi:10.1038/s41419-023-06070-x)
Supplement: Supplementary file 5 — Supplementary figure legend [file 41419_2023_6070_MOESM5_ESM.docx]

**Supplementary figure legends**

**Fig. S1. Resistance to ferroptosis by *KEAP1* mutations is the most prominent feature of lung cancer.** (A) Distribution of drug sensitivity against individual FINs (ML162, ML210, and erastin) in NSCLC cell lines grouped by mutation status of the indicated genes (*KEAP1*, *KRAS*, *TP53*, and *EGFR*). (B-C) Frequencies of *KEAP1* mutations observed in (B) CCLE cancer cell lines across and (C) TCGA patients across different cancer types. (D) FIN sensitivity of pan-cancer cell lines, excluding NSCLC cells, grouped by *KEAP1* mutation status. (E) Statistical significance of the FIN sensitivity differences between *KEAP1* mutant and wild-type cells in NSCLC (black) or in other cell types (grey). The *p*-value was estimated using the Student's t-test to assess statistical significance. (F) mRNA expression levels of key ARE genes in pan-cancer cell lines grouped by *KEAP1* mutation status.

**Fig. S2. Ferroptosis resistance is associated with the expression of FSP1.** (A) Gene ranking by Spearman correlation with the indicated FIN sensitivity based on their mRNA (upper) or protein (lower) expression levels across pan-cancer cell lines. (B-C) Correlation between the indicated FIN (RSL3, ML162, ML210, or erastin) sensitivity and FSP1 abundance (mRNA level, upper; protein level, lower) in (B) pan-cancer cell lines and (C) NSCLC cell lines.

**Fig. S3. iFSP1 treatment makes *KEAP1* mutant cells more sensitive to cysteine depletion-induced ferroptosis.** (A and B) Microscopic morphology of H1299 and A549 cells cultured in cystine-deficient medium in the presence of iFSP1 (3 μM) and Fer-1 (2 μM) for 48 h. iFSP1 and Fer-1 were treated simultaneously upon replacement with cystine-deficient medium. The white line represents a 100 μm scale bar.

**Fig. S4. Inhibition of FSP1 is sufficient to abolish ferroptosis resistance without NRF2 knockdown.** (A) Normalized gene expression levels of NRF2 and its known targets (FSP1, NQO1, FTH1, and HMOX1) in A549 cells with or without NRF2 depletion. Expression data sets were obtained from two independent studies with GEO accession numbers GSE94393 (54) and GSE38332 (53). (B) Western blot analysis showing NRF2, GPX4, and FSP1 in A549 and H460 upon NRF2 knockdown for 48 h (C) Cell death measured by the LDH release of A549 and H460 cells transfected with siNRF2 for 48 h, followed by the treatment with RSL3 (1 μM) and iFSP1 (3 μM) for 24 h. The data are shown as the mean ± S.D (n = 3 independent experiments). The *p*-value is significant for *p* < 0.05 and the measurement of the value was according to Student's t-test.
